# Useful Hints

**Published:** 1874-09

**Authors:** 


*Useful Hints.*—The following is from one who, although too modest to allow his name to be attached, is nevertheless well qualified to judge of the usefulness of anything that pertains to our art:

"I am indebted to my friend Dr. James F. Thompson, the untiring secretary of the Southern Dental Association, for a method of cleaning old files, which will, I am sure, please all who try it. The process is simply to immerse the files for twenty-four hours in a solution of concentrated lye; on removing them, a brush will render them as clean and bright as if new. Of course both the files and the hands must be thoroughly washed to free them from the caustic preparation.

To the same gentleman I am indebted for the suggestion that the ordinary bread soda constitutes one of the very best articles for cleansing the hands that can be used. A trial will convince any one of its merits." H.

